# Primitive neuroectodermal tumor of the kidney in an adult: a case report

**DOI:** 10.4076/1757-1626-2-6791

**Published:** 2009-06-05

**Authors:** Adrian Businger, Andreas Zettl, Stefan Sonnet, Robin Ruszat, Markus von Flüe

**Affiliations:** 1 Department of Surgery, St. ClaraspitalKleinriehenstrasse 30, CH-4016 BaselSwitzerland; 2 Pathology ViollierJacob Burckhardt-Strasse 86, Postfach, CH-4002 BaselSwitzerland; 3 Department of Radiology, St. ClaraspitalKleinriehenstrasse 30, CH-4016 BaselSwitzerland; 4 Urology Unit, St. ClaraspitalKleinriehenstrasse 30, CH-4016 BaselSwitzerland

## Abstract

**Introduction:**

Primitive neuroectodermal tumors (PNETs) occur predominantly in childhood preferentially in the soft tissues of the lower extremity and the paraspinal region. We present here a rare case of a PNET of the kidney in an adult.

**Case presentation:**

A tumor adjacent to the right kidney was detected by ultrasound coincidentally at a routine check-up in a 46-year-old woman with irritable bowel syndrome in her medical history. The patient had no clinical signs. Contrast-enhanced computerized tomography scan of the abdomen demonstrated a highly vascularized renal tumor. A retroperitonealectomy with en-bloc resection of the kidney was performed, and histopathological work-up showed a primitive neuroectodermal tumor of the kidney with the characteristic translocation t(11;22)(q24;q12).

**Conclusion:**

This tumor entity must be accurately distinguished from other renal neoplasms because of the prognostic and therapeutic impact.

## Introduction

Small round cell tumors of the kidney encompass a broad range of neoplasms including lymphoma, clear cell sarcoma of the kidney, neuroblastoma, monophasic Wilms' tumor, carcinoid, desmoplastic small round cell tumor, synovial sarcoma and extraskeletal Ewing sarcoma/ primitive neuroectodermal tumor (ES/PNET) with overlapping morphologic features but different therapeutic regimes. The latter is a rare neoplasm with early regional and distant (lungs, liver, bone) metastasis [1]. The prognosis is poor with a 5-year disease-free survival rate of about 50% despite multimodal therapy [2]. The diagnosis and differential diagnosis is based on immunohistochemical phenotypes and molecular studies [3]. In 1994, Mor described the first case of a ES/PNET in the kidney [4].

Here, we report another patient with a primary renal ES/PNET.

## Case presentation

**Figure 1.**
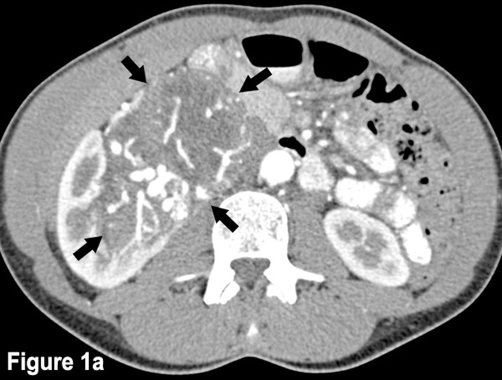
Contrast-enhanced CT scan of the abdomen shows a well-defined, highly vascularized renal tumor. The mass extends towards the renal pelvis and the right kidney is laterally displaced. Tumor borders are indicated by arrows.

**Figure 2.**
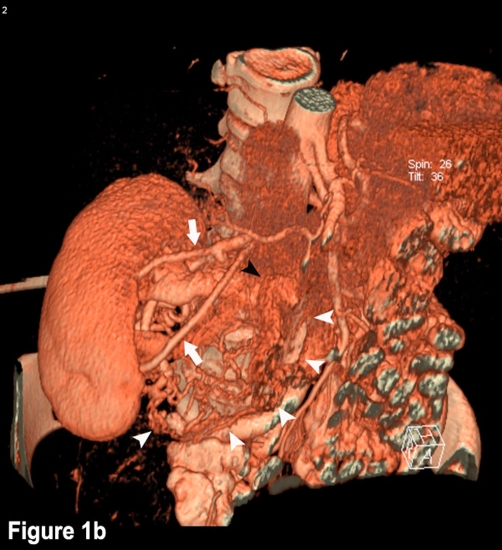
CT angiography of the abdomen at arterial phase shows an extensive tumor vascularization with feeding and draining vessels and arteriovenous shunt formation: Feeding renal arteries (white arrows). Main draining vein (black arrowhead). Pathological vessels (white arrowheads).

During medical examination on the occasion of a routine sonography, an asymptomatic tumor directly near the right kidney and with possible renal origin was detected in a 46-year-old Caucasian woman and Swiss citizen with a history of former smoking with 10 pack-years, irritable bowel syndrome with recurring mild abdominal pain, and a past fibroadenoma excision in the right breast five years ago. The patient complained neither of micturition symptoms nor of fever or feebleness. She had no history of weight loss, but she suffered from night sweats for one month. She had no medication history, and physical findings and laboratory tests were normal. Contrast-enhanced CT scan of the abdomen showed a highly vascularized, well-defined renal tumor with dimensions of 45 × 65 × 102 mm. The mass extended towards the renal pelvis and the right kidney itself was therefore laterally displaced. There was an apparent infiltration of the renal parenchyma or the urinary tract collection system (Figure 1). The 3D-reconstruction of abdominal CT angiography at arterial phase showed an extensive tumor vascularization with feeding and draining vessels and arteriovenous shunt formation (Figure 2). Abdominal MRI confirmed the tumor with an extensive vascularization. We performed an open retroperitonealectomy with removal of the right kidney en-bloc with a caudo-cranial-mobilization of the tumor and several ligations of the ovarian vein, the duplicated renal artery, and the likewise duplicated renal vein. The tumor showed extremely dilated veins on its surface.

Macroscopically, the nephrectomy specimen measured 15 × 12 × 6 cm and weighted 468 g. Almost the whole kidney was infiltrated by a tan to grey, soft tumor mass with multiple foci of necrosis. The tumor had spread to the renal hilus with encasement of the renal artery and vein; the perirenal fatty tissue was free of tumor. The resection margins were free of tumor.

**Figure 3.**
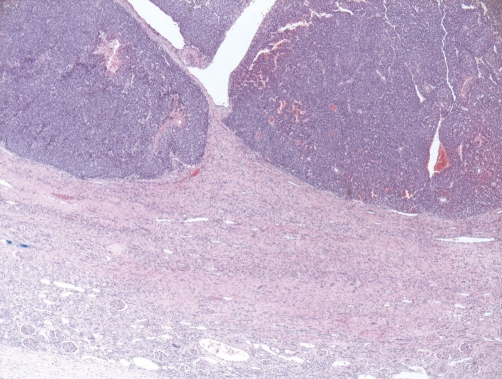
At scanning magnification (HE stain, ×40), a highly cellular neoplasm is seen infiltrating the kidney.

**Figure 4.**
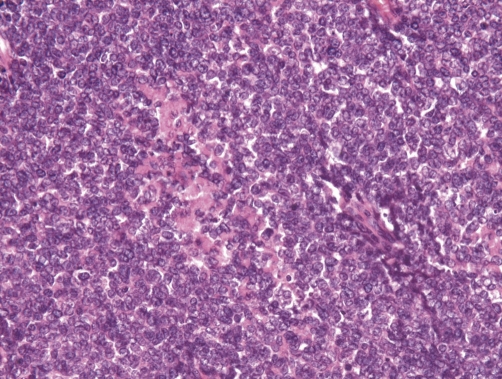
At higher magnification (×200), the neoplasm appears to be composed of tumor cells with round to oval nuclei, very small nucleoli, smoky chromatin, slightly irregular nuclear membranes.

**Figure 5.**
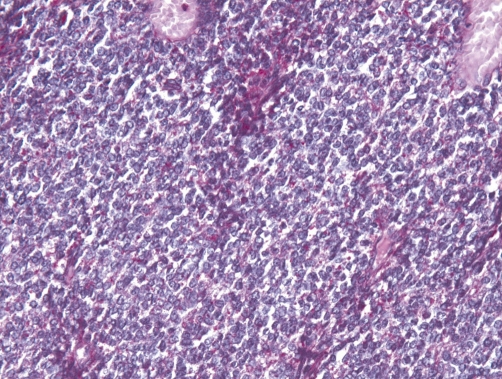
At higher magnification (×200), the neoplasm appears to be composed of tumor cells with round to oval nuclei, very small nucleoli, smoky chromatin, slightly irregular nuclear membranes and small, intensely PAS+ cytoplasm.

Histological examination revealed a uniform population of tumor cells with round to oval nuclei, very small nucleoli, smoky chromatin, slightly irregular nuclear membranes and small, intensely PAS+ cytoplasm (Figure 2c). Numerous signs of mitosis and scattered apoptosis as well as geographic zones of necrosis were encountered (Figures 3-5).

**Figure 6.**
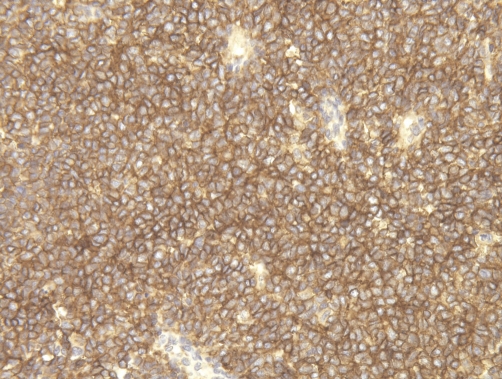
The tumor cells strongly express CD99 (×200).

Immunohistochemical stainings were performed using the automated Bond-maX system (Vision Biosystems, Novocastra). The tumor cells showed strong positivity for CD99 (1:200, Novocastra) (Figure 6), vimentin (1:1000, Dako) and NSE (neuron specific enolase, 1:200, Novocastra). Immunostainings for cytokeratin AE1/3 [(1:800, Dako), EMA (1:50, Dako), S100 (1:24000, Dako), LCA (1:800, DAKO)], CD117 (1:50, Dako), and CD34 (1:400, Dako) were negative.

In molecular studies (performed by L. Guillou, University of Lausanne, Switzerland), the tumor showed the characteristic translocation t(11;22)(q24;q12) with a EWSR1/FLI1 fusion transcript. A diagnosis of primary ES/PNET of the kidney was rendered [4].

Postoperatively, the patient recovered well and was discharged from the hospital after 14 days. She was treated according to the EURO/E.W.I.N.G. 99-protocol with one course of VAI (vincristine, actinomycin D and ifosfamide), followed by seven chemotherapy courses according to the FVAC schema (vincristine, actinomycin and Endoxan).

## Discussion

ES/PNETs of the kidney are extremely rare disease entities [5] and are morphologically and immunophenotypically indistinct from extrarenal ES/PNET [6]. They show the same gene fusions [7] and have a similar poor outcome. In 1994, Mor described the first case of a renal ES/PNET [4]. Subsequently, several case reports and small series of renal PNET were reported in the literature [6,8-10]. Reportedly, renal ES/PNET usually arises in young adults and shows a slight male predominance. Clinically, the tumors usually behave aggressively with frequent metastasis and tumor-related death. In our case, the tumor was asymptomatic and only detected coincidentally. Histologically, several other small round cell tumors enter into the differential diagnosis of renal ES/PNET. Morphological, immunophenotypical and molecular studies are needed to exclude a blastema-predominant Wilms' tumor (which may show areas of stromal or epithelial differentiation, is rarely CD99 positive but frequently shows WT1 expression), metastatic neuroblastoma and clear cell sarcoma (which usually arise in younger patients), synovial sarcoma (which commonly expresses cytokeratin or EMA and shows the characteristic t(X;18)), and small cell carcinoma and lymphoma (which show completely different immunophenotypes). Nonuniform nomenclature of the entity “renal ES/PNET” may have led to underreporting of this entity [11].

## Conclusion

Renal ES/PNET is rare. Differentiation of small round cell tumors of the kidney may be challenging, and asymptomatic lesions in this location are often detected only by chance. In spite of surgical removal and the application of chemotherapy in this chemosensitive tumor with the same nosologic form as Ewing's sarcoma [12], disease-free survival is poor.

## List of abbreviations

CT: computed tomography
ES: Ewing sarcoma
MRI: magnetic resonance imaging
NSE: neuron specific enolase
PNET: primitive neuroectodermal tumor
